# Docosahexaenoic Acid Inhibits Osteoclastogenesis via FFAR4-Mediated Regulation of Inflammatory Cytokines

**DOI:** 10.3390/molecules30153180

**Published:** 2025-07-29

**Authors:** Jinghan Ma, Hideki Kitaura, Fumitoshi Ohori, Aseel Marahleh, Ziqiu Fan, Angyi Lin, Kohei Narita, Kou Murakami, Hiroyasu Kanetaka

**Affiliations:** 1 Division of Orthodontics and Dentofacial Orthopedics, Tohoku University Graduate School of Dentistry, 4-1, Seiryo-machi, Aoba-ku, Sendai 980-8575, Miyagi, Japan; ma.jinghan.c1@tohoku.ac.jp (J.M.); fumitoshi.ohori.b4@tohoku.ac.jp (F.O.); fan.ziqiu.q1@dc.tohoku.ac.jp (Z.F.); lin.angyi.r5@dc.tohoku.ac.jp (A.L.); kohei.narita.a2@tohoku.ac.jp (K.N.); kou.murakami.b2@tohoku.ac.jp (K.M.); hiroyasu.kanetaka.e6@tohoku.ac.jp (H.K.); 2 Frontier Research Institute for Interdisciplinary Sciences, Tohoku University, Sendai 980-8575, Miyagi, Japan; marahleh.aseel.mahmoud.t6@alumni.tohoku.ac.jp; 3 Division of Advanced Dental Science and Technology, Graduate School of Biomedical Engineering, Tohoku University, 6-6-12, Aramaki Aza Aoba Aoba-ku, Sendai 980-8579, Miyagi, Japan

**Keywords:** docosahexaenoic acid, FFAR4, osteoclast, bone metabolism

## Abstract

Osteoclastogenesis—the activation and differentiation of osteoclasts—is one of the pivotal processes of bone remodeling and is regulated by RANKL/RANK signaling, the decoy function of osteoprotegerin (OPG), and a cascade of pro- and anti-inflammatory cytokines. The disruption of this balance leads to pathological bone loss in diseases such as osteoporosis and rheumatoid arthritis. FFAR4 (Free Fatty Acid Receptor 4), a G protein-coupled receptor for long-chain omega-3 fatty acids, has been confirmed as a key mediator of metabolic and anti-inflammatory effects. This review focuses on how FFAR4 acts as the selective receptor for the omega-3 fatty acid docosahexaenoic acid (DHA). It activates two divergent signaling pathways. The Gαq-dependent cascade facilitates intracellular calcium mobilization and ERK1/2 activation. Meanwhile, β-arrestin-2 recruitment inhibits NF-κB. These collective actions reshape the cytokine environment. In macrophages, DHA–FFAR4 signaling lowers the levels of TNF-α, interleukin-6 (IL-6), and IL-1β while increasing IL-10 secretion. Consequently, the activation of NFATc1 and NF-κB p65 is profoundly suppressed under TNF-α or RANKL stimulation. Additionally, DHA modulates the RANKL/OPG axis in osteoblastic cells by suppressing RANKL expression, thereby reducing osteoclast differentiation in an inflammatory mouse model.

## 1. Introduction

Bone is a vital organ involved in movement, body protection, hematopoiesis, and the maintenance of mineral homeostasis [1]. Bone remodeling, which is crucial for maintaining bone health and orthodontic tooth movement (OTM), depends on the balance between osteoblast-mediated bone formation and osteoclast-mediated bone resorption. Osteoclasts, which are characterized by their multinucleated morphology and critical bone-resorbing function, are derived from myeloid precursors that differentiate in situ within the bone marrow [2]. The differentiation and activity of osteoclasts are tightly controlled by macrophage colony-stimulating factor (M-CSF) and receptor activators of nuclear factor-κB ligand (RANKL) [3]. M-CSF promotes precursor cell proliferation, survival, and the expression of RANK, thus sensitizing these cells to RANKL stimulation [4]. The binding of RANKL—produced by osteoblasts, osteocytes, and activated T cells—to RANK initiates a cascade of signaling events involving the recruitment of TRAF6 and the activation of mitogen-activated protein kinases (MAPKs) and nuclear factor-κB (NF-κB), including ERK, JNK, p38, and calcium-dependent pathways [5]. These signaling events culminate in the induction of the nuclear factor of activated T cells cytoplasmic 1 (NFATc1), the master transcription factor for osteoclast differentiation, through the activation of osteoclast-specific genes, such as tartrate-resistant acid phosphatase (TRAP), cathepsin K, and matrix metalloproteinase-9 (MMP-9), thereby allowing for cell fusion and initiating bone resorption activity [6,7,8].

Importantly, osteoclastogenesis is modulated by osteoprotegerin (OPG), a soluble decoy receptor synthesized by osteoblasts and stromal cells, through binding RANKL with high affinity and preventing its interaction with RANK [9]. OPG effectively inhibits the downstream activation of NF-κB and MAPK signaling by sequestering RANKL, thus preventing excessive bone resorption [10,11]. The RANKL/OPG ratio in the bone microenvironment thus plays a key role in determining whether bone resorption or bone formation dominates [12].

Moreover, osteoclastogenesis is dynamically regulated by an intricate network of immune cytokines, linking bone metabolism and systemic and local inflammation. Proinflammatory cytokines, such as interleukin-1β (IL-1β), TNF-α, IL-17, and IL-6, promote osteoclast differentiation both indirectly by promoting RANKL production in stromal cells and osteoblastic cells and directly by making osteoclast precursors more responsive [13]. Specifically, TNF-α increases RANK expression on osteoclast precursors and synergizes with RANKL signaling to promote NF-κB and MAPK pathways, and it can even induce osteoclastogenesis through TRAF2 (TNF receptor-associated factor 2)-mediated signaling when supplemented with appropriate factors [14]. Similarly, IL-1β and IL-6, particularly in inflammatory contexts like rheumatoid arthritis, stimulate osteoclast formation via increased RANKL secretion and direct precursor activation [15,16]. IL-17, produced predominantly by Th17 cells, induces RANKL expression in osteoblastic and stromal cells and cooperates with IL-1β and TNF-α to further activate osteoclast differentiation [17]. In contrast, anti-inflammatory cytokines such as IL-4, IL-10, and transforming growth factor-β (TGF-β) antagonize osteoclastogenesis through the suppression of RANKL expression or interference with precursor differentiation pathways, making the fine-tuning of bone metabolism in physiology and pathology even more complex [18,19,20].

ω-3 fatty acids, which are the active components of fish oil, possess significant health benefits in numerous diseases, including Alzheimer’s disease, cardiovascular disease, depression, type 2 diabetes, age-related macular degeneration, and bone diseases [21]. In vivo and in vitro studies also have found that ω-3 fatty acids help prevent pathological calcification, including both vascular calcification and tumor-associated microcalcifications [22,23,24]. These fatty acids may also improve bone quality by preventing osteoporosis and promoting bone mineralization [25]. The effects of ω-3 FAs depend not only on the types of tissue but also on different molecular mechanisms that suppress pathological calcification in various tissues and support skeletal health [26]. Recently, studies have indicated the benefits of omega-3 fatty acids for bone health, and among them, DHA and EPA play crucial roles. Evidence suggests that DHA exerts more effective inhibitory effects on bone resorption and osteoclast formation than EPA, such as reducing NF-κB activation and TNF-α secretion in macrophages [27,28]. Specifically, DHA also promotes the secretion of IL-10 which is an anti-inflammatory cytokine. Some studies also indicate that DHA can prevent bone loss induced by ovariectomy in mice [29].

Among omega-3 PUFAs, DHA exhibits varied biological activities due to its unique molecular structure. It is characterized by a longer carbon chain and a higher degree of unsaturation for effective incorporation into cell membranes and high signaling capabilities [30]. DHA demonstrates superior anti-inflammatory efficacy compared to EPA [28], notably in reducing proinflammatory cytokine secretion, such as TNF-α and IL-1β, while simultaneously inducing the production of anti-inflammatory cytokines [31,32,33]. Preclinical studies show that DHA significantly inhibits osteoclast differentiation and bone resorption, primarily by disrupting RANKL-induced NF-κB and NFATc1 activation pathways [34]. These effects shed light on the therapeutic feasibility of DHA in diseases associated with excessive bone loss, including osteoporosis, rheumatoid arthritis, and periodontal disease [35].

G protein-coupled receptors (GPCRs) represent the largest and most diverse family of membrane receptors [36] that mediate critical physiological activity by detecting and responding to extracellular ligands, including hormones, neurotransmitters, and dietary fatty acids [37]. Many studies have identified that GPCRs sensitive to omega-3 fatty acids, such as FFAR1 (GPR40) and FFAR4 (GPR120), have broadened the understanding of lipid signaling mechanisms in various biological systems. Among these, FFAR4 has received much attention due to its strong response to DHA and EPA [38]. It was originally known as GPR120 but was renamed FFAR4 after the discovery of long-chain free fatty acids as its primary endogenous agonists [39]. Upon DHA binding, FFAR4 activation triggers two distinct pathways: the Gαq-dependent pathway, which leads to intracellular calcium mobilization and ERK1/2 activation, promoting anti-inflammatory cytokines, and the β-arrestin-2 pathway, which suppresses proinflammatory NF-κB signaling. In bone metabolism, FFAR4 activation has been shown to suppress osteoclast differentiation by inhibiting the expression of osteoclastogenic cytokines and key transcription factors such as NFATc1 [40]. Concurrently, it enhances osteoblast-mediated bone formation, potentially through the modulation of the RANKL/OPG ratio. Thus, DHA-FFAR4 signaling is considered an impactful mechanism that suppresses bone resorption and stimulates bone formation, making FFAR4 an attractive therapeutic target in bone metabolic disorders such as osteoporosis, rheumatoid arthritis, and inflammatory osteolysis [41].

FFAR4 is expressed in numerous tissues, including the gastrointestinal tract, immune cells, adipose tissue, and bone-metabolism-related cells, which regulate inflammation and energy homeostasis. Studies have shown that FFAR4 can also be a therapeutic target to regulate blood glucose levels and increase tissue insulin sensitivity [42]. It regulates metabolic processes, including inflammation, insulin sensitivity, and lipid metabolism [27,43]. Upon activation by its ligands, FFAR4 triggers downstream signaling pathways, such as ERK1/2 MAPK activation and NF-κB suppression, which are involved in anti-inflammatory responses [44]. Additionally, FFAR4 has been implicated in the control of glucose metabolism, with some studies suggesting its potential to improve insulin sensitivity and prevent metabolic disorders such as type 2 diabetes [45]. While FFAR4 has been extensively studied, the overall landscape of its physiological roles and therapeutic potential, along with its involvement in bone metabolism, remains the subject of continuing research.

## 2. Molecular Mechanisms of DHA–FFAR4 Interaction

FFAR4 is a Gαq/11-coupled receptor that responds selectively to long-chain unsaturated fatty acids, with DHA as its most potent endogenous agonist [46,47,48,49]. Upon binding DHA, FFAR4 activates two complementary anti-inflammatory signaling cascades:

### 2.1. DHA–FFAR4 Activation of Gαq-PLCβ/IP_3_-Ca^2+^-ERK1/2 Pathway

DHA binds FFAR4, coupling the receptor to Gαq/11 and activating phospholipase Cβ (PLCβ). PLCβ breaks down PIP_2_ into IP_3_, which stimulates the release of Ca^2+^ from intracellular stores, and Diacylglycerol (DAG), which activates protein kinase C (PKC). The rise in cytosolic Ca^2+^ and PKC activity results in the robust phosphorylation of ERK1/2 MAPK within minutes of DHA stimulation, leading to the expression of anti-inflammatory mediators such as IL-10 [36]. This signaling pathway primarily depends on FFAR4, as the genetic ablation or pharmacological inhibition of FFAR4 eliminates DHA-induced ERK1/2 phosphorylation and subsequent IL-10 production. Additionally, FFAR4 localization in membrane microdomains enhances the coupling efficiency of the receptor to Gαq/11, underscoring the central role in organizing this acute anti-inflammatory response [50,51]. Interestingly, exercise-induced muscle contraction also produces a rapid rise in intracellular Ca^2+^, similar to the surge generated by the DHA activation of the FFAR4 Gαq PLCβ IP_3_ pathway [52,53]. We can hypothesize that ω-3 intake and physical activity converge on a common Ca^2+^-driven network linking bone, muscle, and immune regulation, but this possibility still needs further investigation.

### 2.2. DHA–FFAR4-Induced β-Arrestin-2/TAB1 Axis Mediates NF-κB Pathway

Concurrently, ligand-bound FFAR4 undergoes clathrin-mediated internalization and recruits β-arrestin-2. β-arrestin-2 binds the adaptor protein TAB1, preventing its binding to TAK1, thus activating the IκB kinase (IKK) complex [54]. As a result, NF-κB p65 remains in the cytoplasm, and the transcription of proinflammatory cytokines such as TNF-α, IL-6, and IL-1β is suppressed. It is precisely through the ligand-induced activation of FFAR4 that this β-arrestin-2–TAB1 anti-inflammatory signaling pathway is activated. Studies have shown that the absence of FFAR4 markedly compromises the sequestration of TAB1 by β-arrestin-2, thereby allowing for IKK complex activation and promoting NF-κB transcriptional activity. Thus, the FFAR4 expression level and functional integrity are the critical determinants of the anti-inflammatory effectiveness of this pathway [55].

These two branches of signaling repolarize the cytokine milieu toward resolution by reducing TNF-α and IL-6 secretion in macrophages obviously and also doubling the level of IL-10. In osteoclast precursors, RAW264.7 cells, and primary murine bone marrow, DHA attenuates RANKL-driven NF-κB p65 and NFATc1 activation and reduces TRAP-positive multinucleated cell formation. This function was eliminated in FFAR4-deficient cells [37].

In addition to the above two classical pathways, recent studies have shown that FFAR4 can activate multiple signaling pathways to further regulate osteoclastogenesis. First, FFAR4 activation inhibits RANKL-induced osteoclastogenesis by decreasing intracellular ROS through the upregulation of the Nrf2-HO-1 antioxidant program. Second, ligand-bound FFAR4 can recruit CaMKKβ to activate AMP-activated protein kinase (AMPK) and initiate autophagy; this AMPK-mTOR axis limits osteoclastic differentiation in response to metabolic or glucocorticoid stress in mesenchymal stromal cells and macrophages. Third, macrophage and cancer cell research indicates that FFAR4 intersects with the PI3K-Akt pathway and interacts with NF-κB and mTOR signaling, which may affect osteoclast survival and function. Collectively, these auxiliary pathways highlight the diversity of FFAR4 signaling [56,57,58].

The expression of FFAR4 varies significantly across the different cell types of the bone microenvironment, indicating functional specificity in regulating bone metabolism. Transcriptomic analysis revealed that FFAR4 expression was significantly elevated in late osteoclast differentiation and remained high in osteoblasts, whereas early osteoblast precursors barely expressed the receptor. This distribution pattern suggests that FFAR4 signaling primarily affects the fusion and survival of terminal osteoclasts while regulating osteoblast function [59,60]. Moreover, FFAR4 expression levels are also influenced by different physiological and pathological conditions [61]. Chronic systemic inflammation, aging, and obesity have been reported to downregulate FFAR4 expression in osteoclasts and osteoblasts, which may impair the anti-inflammatory and bone-protective actions of DHA in such conditions [62,63]. Conversely, anti-inflammatory cytokines and some endocrine factors, such as adiponectin, were shown to increase FFAR4 expression, thereby possibly enhancing its therapeutic response [64]. Understanding these regulatory processes is important for the optimization of FFAR4-targeted therapy in various patient groups and disease states.

## 3. DHA Regulation of Immune Cytokines via FFAR4 Through Multiple Pathways

DHA’s anti-osteoclastogenic activity stems from its ability to modulate both pro- and anti-inflammatory cytokines through FFAR4 signaling. Before examining how these pathways alter cytokine networks and osteoclastogenic cues, it is important to recognize that the dual signaling branches—Gαq-mediated PLCβ→Ca^2+^/ERK1/2 activation and β-arrestin-2–dependent NF-κB inhibition—serve as the molecular foundation for all downstream effects. These cascades reshape how macrophages and osteoclast precursors respond to signals by simultaneously promoting anti-inflammatory gene expression and blocking the transcription of TNF-α, IL-6, and IL-1β. This shift establishes a bone microenvironment that restrains osteoclast differentiation.

### 3.1. FFAR4-Mediated Suppression of Proinflammatory Cytokines

In multiple experimental systems, DHA–FFAR4 signaling markedly inhibits proinflammatory cytokine production and osteoclastogenesis. In an LPS-induced inflammation model, Kishikawa et al. showed that daily injections of 100 µg DHA reduced serum TNF-α levels and prevented osteoclast formation in wild-type mice. These effects were absent in FFAR4 knockout mice [65]. Similarly, our previous studies demonstrated that DHA treatment reduced osteoclast numbers and alveolar bone loss in wild-type mice, whereas FFAR4-deficient mice experienced no such protection in an orthodontic tooth movement model [34]. Complementing these in vivo findings, Rahman et al. reported that 20 µM DHA applied to RANKL-stimulated RAW264.7 macrophage-like cells attenuated NF-κB p65 nuclear translocation and NFATc1 upregulation by 60–70%, leading to a corresponding decrease in TRAP-positive multinucleated osteoclasts [55].

### 3.2. FFAR4-Mediated Anti-Inflammatory Polarization

The activation of FFAR4 by DHA not only suppresses proinflammatory signals but also actively promotes anti-inflammatory phenotypes in both macrophages and T cells [27,66,67,68]. In macrophages, DHA-FFAR4 engagement recruits β-arrestin-2 to sequester TAB1, blocking TAK1-IKK and NF-κB activation, while its Gαq-PLCβ-Ca^2+^/ERK1/2 branch doubles IL-10 secretion, driving an M2-like state. In models of intestinal inflammation, FFAR4 agonism enhances IL-10 production by CD4^+^ T cells: treatment with the selective agonist CpdA upregulates Blimp1 and glycolysis via mTOR, increasing IL-10 and protecting mice from Dextran sulfate sodium (DSS)-induced colitis, whereas GPR120-deficient T cells exacerbate disease. In murine colitis models, Salaga et al. showed that the intraperitoneal administration of the selective FFAR4 agonist GSK137647 (1 mg kg^−1^, twice daily) ameliorated DSS- and TNBS-induced disease, as evidenced by reduced body-weight loss, improved macroscopic and histological scores, and a significant reduction in colonic myeloperoxidase activity, indicating dampened neutrophil-driven inflammation [69]. In a separate DSS model, Zhang et al. reported that GSK137647 treatment redirected intestinal macrophage polarization toward an anti-inflammatory M2 phenotype, with an upregulation of IL-4, IL-10, IL-13, and CD206 and concomitant downregulation of the M1 markers CD86, TNF-α, IL-6. and IL-1β, contributing to mucosal protection [70]. These combined actions—enhanced IL-10, M2 polarization, and reduced proinflammatory cytokines—demonstrate how FFAR4 signaling reprograms immune cells to resolve inflammation, a mechanism that also restrains osteoclastogenic cytokine networks in bone [27].

### 3.3. FFAR4-Mediated Regulation of RANKL/OPG Ratio

Beyond its anti-inflammatory actions, FFAR4 activation rebalances stromal cues by suppressing RANKL and enhancing OPG production, reducing the RANKL/OPG ratio and creating an environment unfavorable for osteoclast differentiation [71]. In osteoblast lineage MC3T3-E1 cells, the n-3 fatty acids DHA and EPA upregulate OPG secretion in an FFAR4-dependent manner, lowering the RANKL/OPG ratio; their impact on RANKL transcription is context-dependent and not consistently observed. FFAR4 is readily detectable in differentiated osteoblasts as well as in osteoclast lineage cells [72]. The activation of FFAR4 by the synthetic agonist GW9508 inhibits RANKL-stimulated NFATc1 induction, IκBα phosphorylation, and JNK phosphorylation. It promotes the caspase-3-mediated apoptosis of mature osteoclasts, establishing FFAR4 as a negative regulator of RANKL signaling and osteoclast survival. Moreover, the royal jelly-derived FFAR4 ligand 10-hydroxy-2-decenoic acid binds FFAR4 on osteoclasts to block RANKL-induced NF-κB activation and NFATc1 upregulation, effectively preventing ovariectomy-induced bone loss in mice [37]. These findings demonstrate that FFAR4 in stromal and osteoclast lineage cells diminishes RANKL signaling, lowers the RANKL/OPG ratio, and restrains osteoclastogenesis—mechanisms lost in FFAR4-deficient models—and this underscores FFAR4′s promise as a therapeutic target in bone-destructive disease [40].

These findings reveal that DHA, acting through FFAR4, reprograms innate immune and stromal cell cytokine networks to suppress osteoclastogenesis. Future work should elucidate how FFAR4 expression levels and downstream effectors vary among bone-resident cell types and whether selective FFAR4 agonists can mimic or amplify DHA’s osteoprotective effects in clinical settings.

## 4. DHA-FFAR4 Signaling in Orthodontic Tooth Movement Model

OTM is achieved through a precisely coordinated mechanical loading-induced bone remodeling process. OTM occurs through bone remodeling, with bone resorption on the compression side and bone formation on the tension side [73]. TNF-α is a key proinflammatory cytokine that contributes to bone metabolism [74,75]. It enhances osteoclast differentiation and activation by stimulating the expression of RANKL in osteoblast lineage and periodontal ligament cells. Consequently, TNF-α facilitates bone resorption at sites of mechanical compression, leading to OTM [76]. Recent studies indicate that OTM distance decreases in aging mice [77] and increases in the hypertension mouse model and micro-osteoperforation mouse model [78,79] due to decreased and increased osteoclast formation, respectively.

This remodeling is largely regulated by local inflammatory reactions induced by cytokines such as TNF-α, RANKL, and IL-1β, which are key factors for osteoclastic differentiation and activation [80]. Recent studies highlight DHA’s role in modulating mechanically induced inflammatory response [81]. DHA administration suppresses local proinflammatory cytokine production by infiltrating macrophages and periodontal ligament cells, reducing osteoclast activity and bone resorption markers [82].

In animal models undergoing experimental tooth movement, the dietary supplementation or local administration of DHA decreases osteoclast number and activity, reducing OTM distance and potentially preventing adverse events such as root resorption or alveolar bone loss. These protective effects of DHA are absent in FFAR4-deficient mice, demonstrating that the beneficial modulation of orthodontic inflammatory responses and osteoclastogenesis by DHA depends specifically on FFAR4 signaling [34].

**Figure 1:**
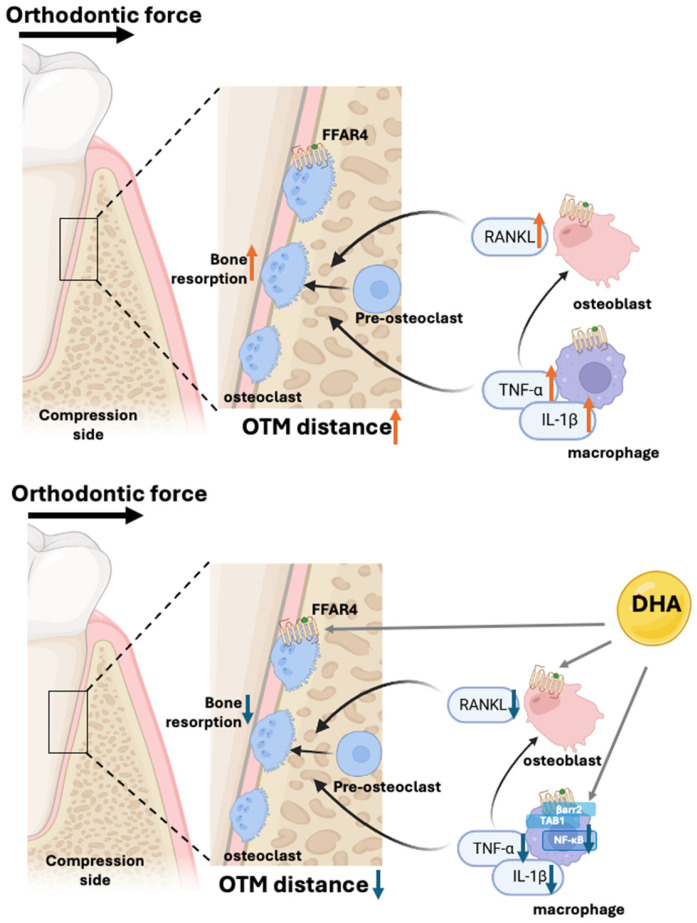
An illustration of the formation of osteoclasts and the role of DHA and FFAR4 in this process. In the absence of DHA, mechanical loading activates macrophages to produce TNF-α and IL-1β (marked in red arrows), which stimulate osteoblast to upregulate RANKL, driving osteoclastogenesis and alveolar bone resorption. With DHA supplementation, FFAR4 was activated, thereby reducing the expression of proinflammatory cytokines and RANKL (marked in blue arrows), reducing osteoclast formation and alveolar bone resorption.

A schematic of how DHA-mediated FFAR4 signaling may influence orthodontic force-induced inflammation and osteoclastogenesis is shown in Figure 1. Given these findings, the DHA–FFAR4 pathway offers potential for improving orthodontic treatments, particularly for patients prone to increased inflammation and reduced bone remodeling, such as those with age-related inflammatory disorders, obesity, or metabolic syndromes. Further clinical studies are needed to apply these findings to clinical orthodontic treatment.

## 5. Preclinical and Clinical Evidence for DHA–FFAR4 in Bone Preservation

### 5.1. Roles of DHA–FFAR4 Signaling on Cellular Level

Several in vitro studies establish that DHA, a crucial omega-3 polyunsaturated fatty acid, regulates osteoclast formation and activity through FFAR4 activation. When DHA binds to FFAR4, it initiates two distinct but complementary signaling pathways. First, DHA-activated β-arrestin-2/TAB1 suppresses NF-κB signaling, reducing TNF-α, IL-6, and IL-1β cytokine secretion. Secondly, DHA triggers the Gαq/PLCβ cascade, increasing intracellular calcium mobilization and ERK1/2 phosphorylation, which promotes IL-10 anti-inflammatory cytokine production. These processes reduce osteoclast precursor sensitivity to RANKL stimulation, NFATc1 activation, and osteoclast-specific gene expression, including TRAP, cathepsin K, and MMP-9. Recent findings highlight that DHA metabolites, resolvin D1 (RvD1) and protectin D1 (PD1), exhibit anti-inflammatory and anti-osteoclastic effects, suggesting DHA’s complex role in regulating bone metabolism [83,84,85].

### 5.2. Roles of DHA–FFAR4 on Animal Models of Bone Diseases

Preclinical studies using animal models consistently support the beneficial role of DHA–FFAR4 signaling in bone-related disorders, including osteoporosis, rheumatoid arthritis (RA), and OTM. In ovariectomy (OVX)-induced osteoporosis models [86], dietary DHA supplementation reduces trabecular bone loss and enhances bone mineral density [87,88,89]. Histological analyses demonstrate reduced osteoclast numbers and suppressed RANKL expression, indicating the direct inhibition of bone resorption. In RA models, DHA administration reduces synovial inflammation, swelling, and erosions. These effects correlate with a decreased local production of inflammatory cytokines and reduced osteoclast activity at inflammatory sites [90,91,92]. Studies on experimental OTM show that DHA supplementation significantly decreases osteoclast formation in periodontal tissues and reduces the risks of mechanically induced alveolar bone resorption. However, these benefits are absent when using FFAR4 knockout mice, providing strong evidence for receptor-specific mechanisms of DHA action [34]. Table 1 presents representative examples of the effects of DHA on bone resorption in cellular, animal models, and disease.

### 5.3. Human Clinical Studies on the DHA–FFAR4 Pathway

Recent clinical studies provide evidence of the beneficial role of DHA–FFAR4 signaling in metabolic and inflammation-related conditions relevant to bone health. In obese and metabolically impaired individuals, DHA supplementation is associated with improved lipid metabolism, including lower serum triglyceride levels and reduced markers of systemic inflammation [96,97,98]. These metabolic benefits are often accompanied by increased insulin sensitivity and decreased bone resorption markers, including CTX-I, suggesting a link between improved metabolism and bone health maintenance [99]. Genetic analyses suggest variability in responses to DHA based on FFAR4 polymorphisms, indicating a personalized aspect of DHA treatment efficacy.

Clinical evidence from human studies confirms the positive impacts of DHA consumption on bone metabolism. Epidemiological studies consistently show an inverse correlation between dietary n-6/n-3 PUFA ratios and bone mineral density (BMD) among older populations [100,101]. Higher DHA intake is associated with improved BMD at key sites, such as the hip and lumbar spine, reducing fracture risk [102]. Clinical trials confirm these observations, reporting that postmenopausal women receiving DHA supplementation exhibit elevated serum osteocalcin and reduced bone resorption markers [103,104].

In patients with rheumatoid arthritis, DHA supplementation provides symptomatic relief, reducing joint pain, swelling, and morning stiffness [105]. Concurrently, decreases in inflammatory biomarkers, such as CRP, TNF-α, and IL-6, are observed, along with radiographic evidence of slowed joint erosion [106]. This dual anti-inflammatory and anti-resorptive effect highlights DHA–FFAR4 signaling as a potential therapeutic avenue in inflammatory joint diseases [107,108,109].

Studies in patients with metabolic syndrome and type 2 diabetes mellitus demonstrate improvements following DHA supplementation, including enhanced lipid profiles, reduced blood pressure, and improved insulin sensitivity [110]. These metabolic improvements likely exert indirect protective effects on bone health by diminishing chronic systemic inflammation, a recognized contributor to osteoporosis and fracture risk [93]. These clinical findings reinforce the therapeutic potential of DHA–FFAR4 signaling as a holistic approach to managing metabolic dysfunctions and bone health [95].

**Figure 2:**
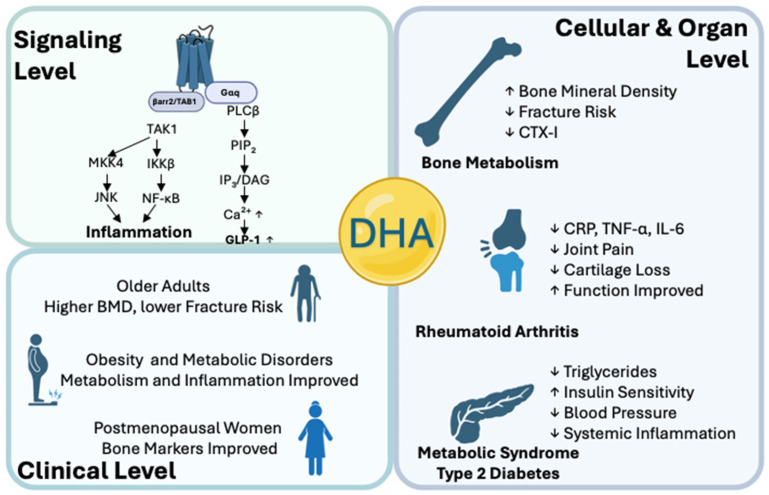
Effect of DHA-activated FFAR4 signaling on inflammatory and clinical benefits, on signaling level, clinical level, and cellular and organ level. Figure created with BioRender.com.

Additionally, emerging clinical evidence points to the potential benefits of DHA–FFAR4 activation in mechanical-stress-induced osteoarthritis. Clinical trials involving daily DHA supplementation for several months report improvements in osteoarthritis symptoms, including reduced joint pain and functional impairment, as assessed by standardized clinical scales. Synovial fluid analyses in these patients demonstrated decreased concentrations of cartilage-degrading enzymes. These findings were supported by radiographic imaging showing slowed cartilage loss progression [41]. At a mechanistic level, investigations on patient-derived chondrocytes show a reduced activation of inflammatory pathways, such as NF-κB with DHA treatment, confirming a direct anti-inflammatory effect mediated by FFAR4 signaling. These human clinical observations indicate that DHA, via FFAR4, could serve as an adjunctive nutritional strategy to counteract inflammatory damage in mechanically compromised joints, preserving cartilage integrity and improving patient outcomes [107]. Figure 2 summarizes DHA-FFAR4 anti-informatory and bone-protective pathways.

## 6. Summary and Future Perspectives

Preclinical data suggest that DHA–FFAR4 activation suppresses osteoclastogenesis, inflammation, and bone resorption through two major mechanistic pathways. First, the activation of the β-arrestin-2/TAB1 pathway inhibits NF-κB signaling, reducing the level of proinflammatory cytokines. Secondly, Gαq/PLCβ-mediated Ca^2+^/ERK1/2 signaling promotes IL-10 release, modulating the cytokine microenvironment toward an anti-inflammatory response [111]. This signaling pathway inhibits RANKL-induced NFATc1 activation and reduces the expression of key osteoclast markers such as TRAP, cathepsin K, and MMP-9. Additionally, DHA metabolites such as RvD1 and PD1 further reinforce these anti-inflammatory and anti-resorptive actions.

Clinical evidence supports these preclinical findings. Epidemiological and clinical studies also show a relationship between high DHA consumption and improved bone density, reduced fracture risk, and alleviated rheumatoid arthritis symptoms, as well as beneficial effects on metabolic syndrome and diabetes mellitus. Therefore, DHA-FFAR4 signaling emerges as a viable target for both bone protection and modulating metabolic disorders. Optimal DHA doses, treatment duration, and administration methods will enhance clinical efficacy and safety for bone-related diseases, particularly in patients with obesity, dyslipidemia, or inflammation-related conditions, such as rheumatoid arthritis and mechanically induced osteoarthritis.

However, clinically effective DHA concentrations in human bone tissue remain undetermined. Variability in FFAR4 expression and response among patient populations, particularly the elderly, obese, and those with chronic inflammation, necessitates further research. The lack of highly selective FFAR4 agonists limits clinical utility and potential for targeted pharmacological development. Concomitant DHA administration and mechanical loading may provide insight into the potential interaction between mechanical loading and FFAR4 stimulation. DHA supplementation, combined with diet-related strategies to reduce systemic inflammation, may improve its therapeutic benefits.

Further research should focus on several areas, including conducting targeted clinical trials to refine DHA dosing regimens and administration methods and elucidating the regulatory mechanisms controlling FFAR4 expression across a diverse population and diseases to enable personalized treatment regimens. Research should also accelerate the development of selective FFAR4 agonists to improve clinical efficacy and define the role of DHA–FFAR4 signaling in OTM and other metabolic bone diseases. These research efforts are expected to lead to evidence-based treatments that integrate DHA–FFAR4 signaling, benefiting patients afflicted with osteoporosis, arthritis, and related metabolic bone diseases.

## 7. Conclusions

This review demonstrated that DHA-FFAR4 simultaneously activates the β-arrestin-2/TAB1-NF-κB inhibitory pathway and the Gαq/PLCβ-Ca^2+^/ERK-IL-10 proinflammatory pathway. This leads to the downregulation of TNF-α, the inhibition of RANKL-NFATc1 signaling, and decreased TRAP and cathepsin K expression, thereby inhibiting osteoclastogenesis and bone resorption. Animal studies suggest that high DHA supplementation increases bone density, significantly attenuates RANKL and TNF-α levels on the compression side of the OTM model, reduces osteoclast numbers, and decreases alveolar bone resorption during OTM. Overall, the DHA-FFAR4 axis may provide a potential therapeutic target for osteoporosis, rheumatoid/osteoarthritis, and OTM-associated bone loss and should be followed up with further clinical experiments and the development of highly selective FFAR4 agonists to achieve precise therapeutic effects.

## Figures and Tables

**Table 1:** This table provides a concise overview of the cell, animal, and human studies mentioned, listing sample size, DHA (or related agonist) dose, dosing frequency, administration route, and the main osteoclast-related or disease-related outcome.

Citation	Model	Treatment Dose	Duration	Effect on Bone Resorption
**Cell** Sithole et al., 2021 [56]	RAW264.7 macrophages	TUG-891 (GPR120 agonist) 20–100 µM	5 days	Inhibits osteoclast activity—blocks RANKL-induced TRAP^+^ multinuclear cells
Kasonga et al., 2019 [71]	RAW264.7 MC3T3-E1	DHA 40 µM	≤5 days	Inhibits osteoclast activity—DHA-activated GPR120/β-arrestin-2 blocks RANKL-NF-κB/MAPK
**Animal** Tsuchiya et al., 2020 [37]	OVX mice (and OC cultures)	In vivo: 10H2DA (GPR120 agonist) 40 mg kg^−1^ In vitro: 500 µM	4 weeks	Inhibits osteoclast activity—10H2DA blocks RANKL-NF-κB/NFATc1, reducing bone loss
Ahn et al., 2016 [59]	In vivo: fat-1 × Ffar4-/- mice (±OVX) with calvarial injection In vitro: RAW264.7 pre-osteoclasts	Endogenously elevated n-3 fatty acids In vivo: DHA 50–250 µM In vitro: DHA 40 µM	4–8 wk experimental periods ≤5 d (culture)	Inhibits bone resorption
Sun et al., 2003 [87]	OVX mice (and BMM cultures)	In vivo: 5% fish oil diet In vitro: DHA/EPA 40 µM	4 months	Inhibits osteoclast activity—prevents trabecular bone loss; suppresses NF-κB and osteoclast formation
Su et al., 2024 [43]	RAW264.7 macrophages and CAIA mice	In vitro: lipid mediator mix from DHA 1 µg mL^−1^ In vivo: 10 µg kg^−1^ day^−1^ oral	5-day culture 10-day dosing	Inhibits osteoclast activity—DHA-derived lipid mediators suppress RANKL-induced TRAP and CTSK via NF-κB and reduce bone erosion
**Human**				
Xiao et al., 2022 [93]	Meta-analysis 46 RCTs, n = 4991	Fish-oil ≤ 2 vs. >2 g d^−1^	4–12 months	Improves lipid profile
Mei et al., 2021 [94]	Prospective cohort, UK 40–70 y, n = 378,018	With or without fish oil supplement	12 years	Reduces fracture risk
Díaz-Rizzolo et al., 2021 [95]	RCT ≥ 65 y, n = 152	Sardine 200 g week^−1^ (≈3 g EPA + DHA)	12 months	Lowers risk of type 2 diabetes

## Data Availability

The data presented in this study are available upon request from the corresponding author.

## Abbreviations

The following abbreviations are used in this manuscript:

FFAR4	Free Fatty Acid Receptor 4
GPR120	G Protein-Coupled Receptor 120
DHA	Docosahexaenoic Acid
EPA	Eicosapentaenoic Acid
RANKL	Receptor Activator of Nuclear Factor-κB Ligand
OPG	Osteoprotegerin
NF-κB	Nuclear Factor Kappa-Light-Chain-Enhancer of Activated B Cells
MAPK	Mitogen-Activated Protein Kinase
NFATc1	Nuclear Factor of Activated T-Cells, Cytoplasmic 1
IL-10	Interleukin-10
TNF-α	Tumor Necrosis Factor-α
LPS	Lipopolysaccharide
OVX	Ovariectomized
TRAP	Tartrate-Resistant Acid Phosphatase
PLCβ	Phospholipase C Beta
TAK1	Transforming Growth Factor β-Activated Kinase 1
TAB1	TAK1-Binding Protein 1
ERK1/2	Extracellular Signal-Regulated Kinases 1/2
OTM	Orthodontic Tooth Movement
JNK	c-Jun N-terminal Kinase
MMP-9	Matrix Metalloproteinase-9
TRAF2	TNF Receptor-Associated Factor 2
DAG	Diacylglycerol
PKC	Protein Kinase C
